# The Involvement of the Serotonergic System in Ketamine and Fluoxetine Combination-induced Cognitive Impairments in Mice

**DOI:** 10.5152/eurasianjmed.2024.23219

**Published:** 2024-06-01

**Authors:** Emre Uyar, Meral Erdinç, İlker Kelle, Levent Erdinç, Uğur Şeker, Yusuf Nergiz

**Affiliations:** 1 Department of Medical Pharmacology, Uskudar University Faculty of Medicine, Istanbul, Türkiye; 2 Department of Medical Pharmacology, Dicle University Faculty of Medicine, Diyarbakir, Türkiye; 3 Department of Biochemistry, Dicle University Faculty of Medicine, Diyarbakir, Türkiye; 4 Department of Histology and Embryology, Mardin Artuklu University Faculty of Medicine, Mardin, Türkiye; 5 Department of Histology and Embryology, Dicle University Faculty of Medicine, Diyarbakir, Türkiye

**Keywords:** Ketamine, serotonergic system, passive avoidance test, emotional memory, brain morphology

## Abstract

**Background:**

Glutamatergic N-methyl-D-aspartate (NMDA) receptors play vital roles in memory formation. Changes in the activity of these receptors influence memory processes. Ketamine is a noncompetitive NMDA receptor antagonist drug with promising mood-altering and pain-reducing effects in low doses. These effects are believed to be related to altered serotonergic transmission.

**Methods:**

The present study investigated the involvement of the serotonergic system in low-dose ketamine administrations’ effects on memory acquisition, consolidation, and retrieval processes. Sixty-four male BALB/c mice were used in this experiment and separated into 8t groups. Mice were treated subchronically with a selective serotonin reuptake inhibitor, fluoxetine, and a serotonin depletion agent, p-chlorophenylalanine (pCPA). A serotonin antagonist, methiothepin, and ketamine were acutely administered 60 minutes before or after the behavioral tests. A passive avoidance (PA) test measured emotional memory acquisition, consolidation, and retrieval processes. Hippocampi malondialdehyde (MDA) levels were analyzed, and histopathological examinations were performed.

**Results:**

Ketamine alone did not significantly affect memory encoding processes in the PA test, while the ketamine–fluoxetine combination disrupted memory consolidation. Fluoxetine negatively affected the memory acquisition process, which was normalized during the consolidation and retrieval trials. Drug applications did not significantly alter hippocampal MDA levels. In all ketamine-applied groups, histopathologic alterations were evident.

**Conclusion:**

Low-dose ketamine administration induces neurodegeneration, and it also impairs memory functions when combined with fluoxetine, indicating increased serotonergic transmission may be involved in the memory-impairing and neurotoxic effects of ketamine.

**Main Points**

- Ketamine alone did not alter emotional memory functions.
- Ketamine combined with fluoxetine impaired memory acquisition and consolidation.
- Fluoxetine alone impaired memory acquisition but not consolidation and retrieval.
- The importance of the serotonergic system is observed in ketamine–fluoxetine combination-induced memory impairments.

## Introduction

Glutamatergic receptors are involved in various cognitive processes, including memory.^1^ Increased glutamatergic transmission can enhance memory functions, while reduced transmission can impair memory.^2,3^ Long-term potentiation and long-term depression are mediated by complex mechanisms involving the glutamatergic N-methyl-D-aspartate (NMDA) receptors.^4^

The normoactivity of NMDA receptors is essential for memory formation, synaptic plasticity, and proper brain function.^5^ NMDA receptor antagonists affect cognition; thus, these molecules in high doses, including ketamine, are being employed to induce memory impairments,^6^ while lower doses are investigated to treat depression,^7^ pain,^8^ and anxiety disorders.^9^ Suicidal ideation in humans is also reduced with low-dose ketamine applications.^10^

Cognitive functions are sensitive to changes in neurotransmission. The serotonergic system is crucial in memory formation, and significant alterations in serotonergic transmission may affect cognitive functions.^11^ Ketamine is reported to increase serotonergic activity, which is deemed to be linked to its fast-acting antidepressant effects.^12^ Similar mechanisms may be involved in its deteriorating effects on memory processes.

Ketamine is associated with neurodegenerative changes in various areas of the brain in preclinical and clinical studies. Loss of hippocampal tissue integrity and loss or degeneration of pyramidal and granule neurons are linked with ketamine applications.^13^ Clinical studies on individuals with ketamine addiction also revealed significant morphological deteriorations in a wide range of brain regions.^14^ Although ketamine has definite benefits in various indications, its memory-impairing and neurotoxic effects limit its widespread use.^15^

The present study was performed to determine the acute effects of low-dose ketamine administrations and ketamine–fluoxetine combinations on memory processes, e.g., acquisition, consolidation, and retrieval, and to define the serotonergic systems’ possible involvement in ketamine-induced memory impairments and neurotoxicity in mice.

## Material and Methods

### Animals

Sixty-four male BALB/c mice (10-12 months of age, weighing 25-35 g) obtained from the local animal colony facility were separated into 8 groups (n = 8). The sample size was determined based on power analysis provided by Inonu University Online Statistics Application calculations, which considered statistical significance, effect size, and anticipated variability to ensure adequate statistical power for detecting meaningful differences between experimental groups. The mice were housed in plastic cages with free access to food pellets and tap water. Two weeks before the experimental procedures, they were transferred to the laboratory and kept in standard laboratory conditions (21 ± 1.5°C) in a 12-hour light/dark cycle (light onset at 8:00 pm). Each test group consisted of 7-9 mice, and all tests were conducted between 9:00 and 12:00 am. All procedures concerning the animals were performed in agreement with Directive 2010/63/EU of the European Parliament and of the Council of 22 September 2010. Ethics approval was obtained from Dicle University Animal Experiments Ethics Committee (DUHADEK) with the number: 1412, Date: 2014. The mice were naive to the conducted protocols and were tested individually. Following the behavioral tests, the mice were sacrificed by cervical decapitation for further analysis. One brain hemisphere was used for histopathological analysis, and the other was used to measure hippocampal malondialdehyde (MDA) levels.

### The Experimental Groups

Eight groups were named as follows: (1) VEH: vehicle, (2) KET: ketamine (20 mg/kg), (3) FLU: Fluoxetine (20 mg/kg, 7 days), (4) MET: methiothepin (0.1 mg/kg), (5) pCPA: para-chlorophenylalanine (150 mg/kg, 4 days), (6) KF: ketamine–fluoxetine combination, (7) KM: ketamine–methiothepin combination, and (8) KP: ketamine–pCPA combination. Combinations had similar application protocols as the single-drug administered groups. Fluoxetine applications started 7 days before the first experiment (passive avoidance acquisition trial) and continued until the end of the experimental procedure. Para-chlorophenylalanine applications were started 4 days before the first experiment and continued until the end. Other molecules (KET and MET) were administered only before or after the experiments, as explained below.

#### Drugs

Drugs were obtained from Sigma-Aldrich Chemical Co. (St. Louis, MO, USA). Fluoxetine (20 mg/kg, 1 week) was used as a selective serotonin reuptake inhibitor; MET (0.01 mg/kg) was used to block serotonin receptors; and pCPA (150 mg/kg, 4 days) was used to deplete serotonin and block its production via inhibiting tryptophan hydroxylase. All drugs were dissolved in 0.9% saline and given intraperitoneally in a volume of 10 mL per kg body weight. The mice received fluoxetine and pCPA before and on testing days, and the untreated groups were given 0.9% saline. The drugs KET (20 mg/kg), MET, and continuously administered drugs (pCPA and fluoxetine) were injected 1 hour before the PA test training (acquisition, day 1), directly after the training (consolidation, day 1), and 1 hour before the retention (retrieval, day 2) trials. The doses of the drugs were determined based on earlier behavioral studies.^16-18^

#### Passive Avoidance Test

A passive avoidance (PA) test apparatus (MAY-PA 1014-M) was used to measure the effects of the drugs on emotional learning and memory performances. In this test, the animals learn to evade a place linked to an unpleasant experience. The apparatus comprised 2 compartments (22 × 21 × 22 cm each). The illumined white compartment (2000 lux) is connected to a dark compartment with an automatically operated ground-level door. The passing time of mice to the dark compartment was considered step-through latencies.^19^ The latencies to enter the dark compartment on the second day of the test were used as an indicator of learning. The apparatus is decorated with an electrifiable grid floor. The test lasted 2 days and consisted of training (day 1) and a retention trial (day 2).

In the training trial, each mouse was individually placed in the illumined compartment and acclimated for 60 seconds, after which the door separating the compartments was opened. When the mouse placed all 4 paws into the dark compartment, the door closed automatically, and a footshock was administered to the animal’s paws (0.25 mA, 1 second). Then, they were removed from the apparatus and returned to their home cage. The mice not entering the dark compartment in 300 seconds (cut-off time) were excluded from further experiments. The grid floor and the small drawer under the apparatus were cleaned thoroughly between each test to not to affect the following mice with olfactory cues. In the retention trial conducted the day following the training, the animals were placed into the illumined compartment, and the step-through latencies were estimated. The latencies to enter the dark compartment in this trial were used as an indicator of learning.

#### Measurement of Lipid Peroxidation

Malondialdehyde is a lipid peroxidation product and is used to measure oxidation levels. The thiobarbituric acid (TBA) method was used to assess MDA levels based on the reaction of MDA with TBA.^20^ The tissue samples were weighed and placed in ice-cold 0.5 mL 10% trichloroacetic acid (TCA), along with 4.5 mL of 5% TCA (w/v). Then, the tissues were homogenized using a sonic dismembrator (Model FB50, Fisher Scientific, California, USA) and centrifuged with a microcentrifuge (MicroCL 17R, Thermo Scientific, Massachusetts, USA) for 15 minutes at 4500 rpm. Following centrifugation, 1 mL of the supernatant was transferred into a glass tube with an equivalent volume of 0.6% (w/v) TBA, and the mixture was heated using a Nuve water bath (BM 30, NUVE, Ankara, Türkiye) and kept at 100°C for 10 minutes. After cooling, the absorption spectrum was measured with a spectrophotometer (Shimadzu UV-1205, Kyoto, Japan) at 532 nm. Results were measured using the molar extinction coefficient and were shown as nmol per gram of tissue.

#### Histopathological Examinations

The brain tissues taken from the mice were placed in a 10% formalin solution for 24 hours for fixation and further pathological analysis, as described previously.^21^ The sections were subjected to routine histological tissue preparation procedures after the fixation. The tissues were dehydrated and embedded in paraffin blocks. Sections of 5 µm thickness were obtained from the paraffin blocks and stained with hematoxylin and eosin (H&E). An observer blinded to the experimental protocol observed the sections under a Nikon light microscope (Nikon Eclipse 80i, Nikon, Tokyo, Japan).

### Statistical Analysis

The results were assessed with SPSS 24 (IBM SPSS Corp.; Armonk, NY, USA) using a one-way analysis of variance (ANOVA). A post hoc Tukey test was used to define the differences between groups. The data are shown as mean values and ±SEM. Differences were considered statistically significant if the *P*-value was less than .05.

## Results

### Passive Avoidance Tests

These tests were executed in 3 different setups to evaluate the effects of drug application protocols on memory acquisition, consolidation, and retrieval processes. Drugs were applied 1 hour before the first-day test to measure memory acquisition, right after the first-day test to measure memory consolidation, and 1 hour before the second-day test to measure memory retrieval.

### Memory Acquisition Trial

**Figure 1.**
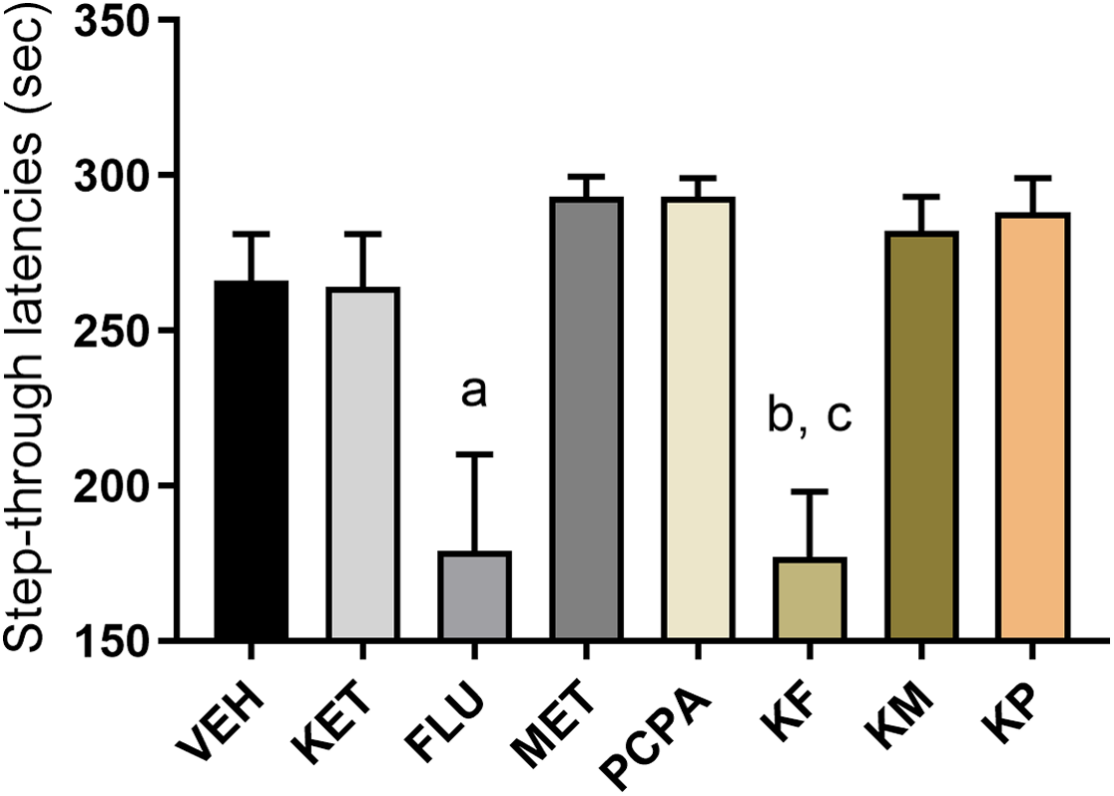
Memory acquisition trial, step-through latencies in the passive avoidance test. VEH, vehicle; KET, ketamine (20 mg/kg); FLU, fluoxetine (20 mg/kg, 7 days); MET, methiothepin (0.1 mg/kg); pCPA, para-chlorophenylalanine (150 mg/kg, 4 days); KF, ketamine–fluoxetine combination; KM, ketamine–methiothepin combination; and KP, ketamine–pCPA combination. Each column represents the mean ± SEM of 7-8 mice. ^a^*P* < .01 vs. VEH group, ^b^*P* < .05 vs. VEH group, ^c^*P* < .05 vs. KET group. One-way ANOVA followed by a post hoc Tukey test was used for statistical analysis.

The step-through latencies were not altered in the KET group but were lower in the FLU and KF groups compared with the VEH group (*P* < .01, *P* < .05, respectively). The KF group’s latencies were also lower than the KET group (*P* < .05, Figure 1).

### Memory Consolidation Trial

**Figure 2.**
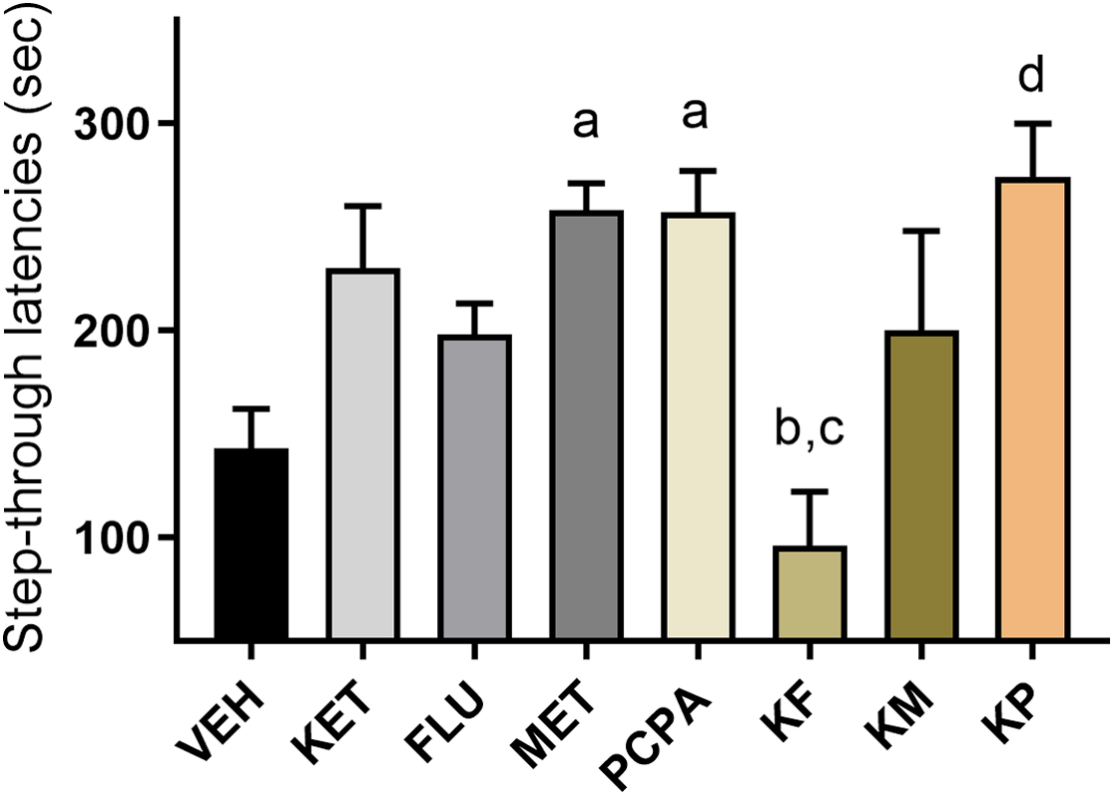
Memory consolidation trial, step-through latencies in the passive avoidance test. VEH, vehicle; KET, ketamine (20 mg/kg); FLU, fluoxetine (20 mg/kg, 7 days); MET, methiothepin (0.1 mg/kg); pCPA, para-chlorophenylalanine (150 mg/kg, 4 days); KF, ketamine–fluoxetine combination; KM, ketamine–methiothepin combination; KP, ketamine–pCPA combination. Each column represents the mean ± SEM of 7-8 mice. ^a^*P* < .05 vs. VEH group, ^b^*P* < .01 vs. KET group, ^c^*P* < .05 vs. FLU group, and ^d^*P* < .01 vs. VEH group. One-way ANOVA followed by a post hoc Tukey test was used for statistical analysis.

The KF group had lower transfer latencies than the KET group (*P* < .01). MET, pCPA, and KP groups had significantly increased step-through latencies compared to the VEH group (*P* < .05 *P* < .05, and *P* < .01, respectively). The KF group had lower step-through latencies than the FLU group (*P* < .05, Figure 2).

### Memory Retrieval Trial

**Figure 3.**
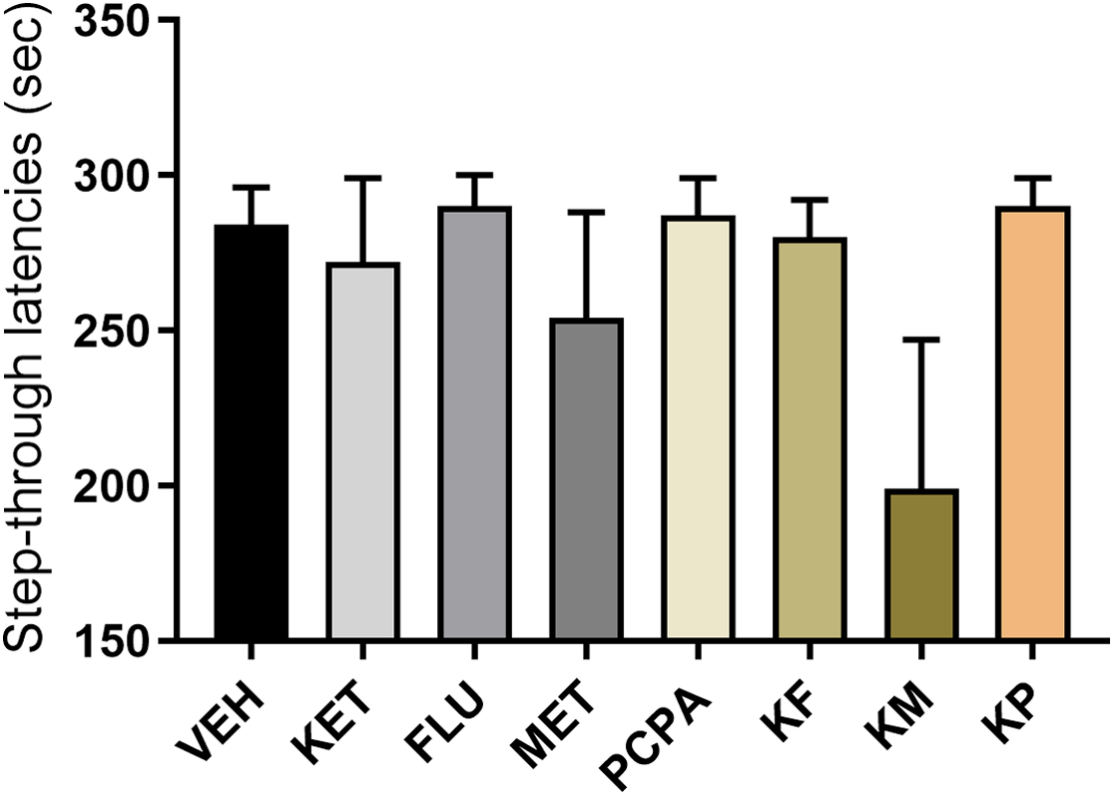
Memory retrieval trial, step-through latencies in the passive avoidance test. VEH, vehicle; KET, ketamine (20 mg/kg); FLU, fluoxetine (20 mg/kg, 7 days); MET, methiothepin (0.1 mg/kg); pCPA, para-chlorophenylalanine (150 mg/kg, 4 days); KF, ketamine–fluoxetine combination; KM, ketamine–methiothepin combination; KP, ketamine–pCPA combination. Each column represents the mean ± SEM of 7-8 mice. One-way ANOVA was used for statistical analysis.

No significant differences were observed between test groups (*P* > .05, Figure 3).

### Histopathological Results

**Figure 4.**
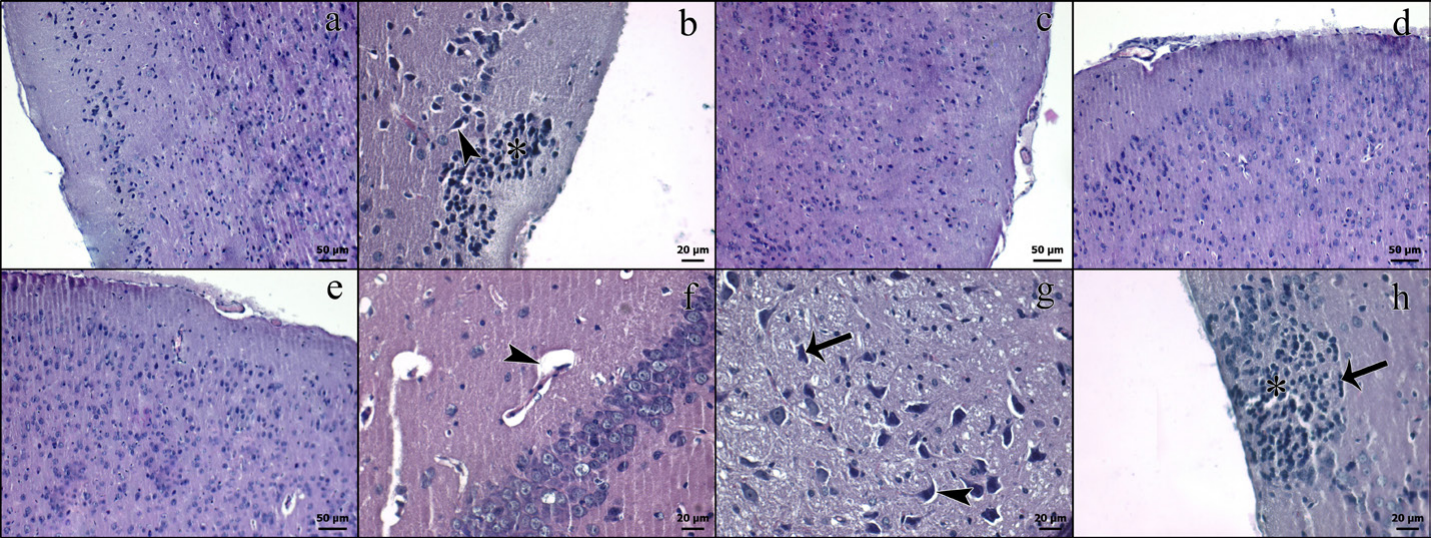
A–H: Histopathologic micrographs of groups. Normal histologic appearance of the brain in VEH (A), FLU (C), MET (D), and pCPA (E) groups. Histopathologic appearances in KET (B), KF (F), KM (G), and KP (H) groups. Arrowhead: perineural and perivascular edema, arrow: necrosis, asterisk: satellitosis. Staining: hematoxylin and eosin, Bar: 50 µm (A, C, D, E), 20 µm (B, F, G, H).

Brain slides were examined by using a light microscope, and normal histologic appearances were observed in the VEH (Figure 4A), FLU (Figure 4C), MET (Figure 4D), and pCPA (Figure 4E) groups. Perineural and perivascular edema were observed in KET (Figure 4B), KF (Figure 4F), and KM (Figure 4G) groups. Necrosis was observed in KM (Figure 4G) and KP (Figure 4H) groups. Satellitosis was observed in KET (Figure 4B) and KP (Figure 4H).

### Lipid Peroxidation Levels

**Figure 5.**
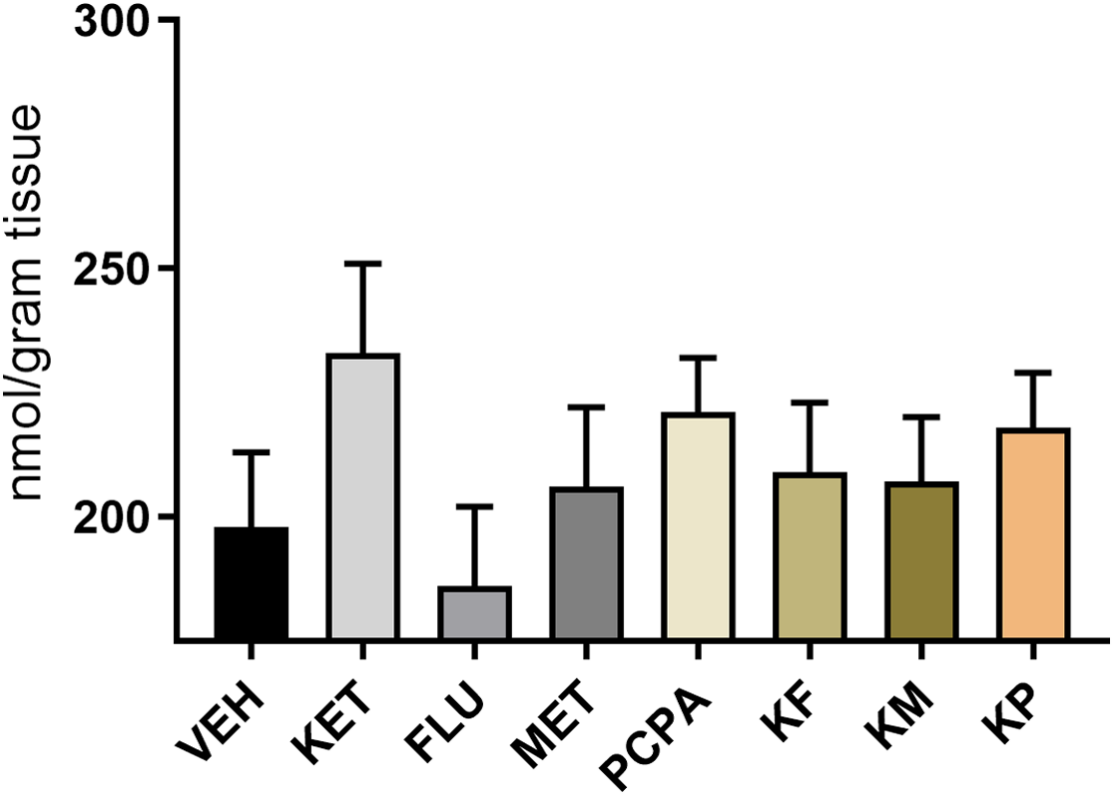
Hippocampal MDA levels of the mice. VEH, vehicle; KET, ketamine (20 mg/kg); FLU, fluoxetine (20 mg/kg, 7 days); MET, methiothepin (0.1 mg/kg); pCPA, para-chlorophenylalanine (150 mg/kg, 4 days); KF, ketamine–fluoxetine combination; KM, ketamine–methiothepin combination; KP, ketamine–pCPA combination. Each column represents the mean ± SEM of 7-8 mice’s hippocampal MDA levels. One-way ANOVA was used for statistical analysis.

Hippocampal MDA levels were insignificantly higher in the KET group compared with the VEH group (*P* > .05, Figure 5).

## Discussion

Ketamine is a popular drug presented as an anesthetic in the second part of the past century. Apart from its anesthetic effects, it has various therapeutic indications, including depression, anxiety, and pain disorders.^8,9^ Apart from its therapeutic effects, ketamine has severe toxicity on memory functions, brain functionality, and morphology.^13^ Thus, revealing ketamine’s effects on memory processes, neurotoxic potential, and underlying mechanisms in these effects is necessary.

Preclinical and clinical studies reported memory-impairing effects related to ketamine applications. Ketamine is reported to impair episodic and working memory, slow semantic processing, and impair recognition memory and procedural learning in humans in a dose-dependent manner.^22^ Although a single dose of ketamine is reported not to alter memory functions, chronic ketamine exposure induces learning and memory deficits.^23,24^ While high doses and prolonged applications cause neurotoxicity,^25,26^ low doses are reported to increase the development of dendritic spines and reduce neurodegeneration.^27^

This study utilized a passive avoidance test to evaluate emotional memory processes (i.e., acquisition, consolidation, and retrieval). In this test, ketamine did not impair these processes when applied alone. A study reported similar results in the mentioned memory processes with single-dose ketamine (40 mg/kg) midazolam combination applications.^23^ Another study reported impaired memory acquisition and retrieval performance after single low- and high-dose ketamine applications (15 mg/kg, 100 mg/kg, respectively); consolidation is affected only by high-dose ketamine.^28^

Interestingly, the combination of ketamine with fluoxetine impaired both memory acquisition and consolidation but not retrieval. Fluoxetine alone also disrupted memory acquisition, but not consolidation, and retrieval. Although fluoxetine is reported to protect against memory impairments, some studies have reported the memory-impairing effects of the drug.^29-31^ A study reported long-term memory impairments with chronic fluoxetine applications, which were restored with drug withdrawal.^32^ Another study reported impaired memory functions with subchronic fluoxetine applications in adolescence, which affected the learning capacity of their adulthood.^33^ The effects of fluoxetine on memory functions seem to be dependent on the application period, time, and dose.

Augmentation of the memory impairment with fluoxetine in the consolidation process that was disrupted in the acquisition may be due to receptor downregulation and desensitization. Ketamine seems to add to the memory-impairing effects of fluoxetine through serotonergic mechanisms. Antiserotonergic drugs, on the other hand, pCPA and MET, improved the consolidation process in this experiment. This improvement may not be related to improved memory functions but to remembering a bad experience induced by an electric shock in the PA test, as the mentioned molecules are reported to produce hyperalgesic effects.^34,35^

Increased oxidative stress is known to cause cell injury. Ketamine is reported to increase oxidative stress and suppress defensive mechanisms, which is believed to contribute to the drug’s neurotoxic effects.^36^ Studies reported increased oxidative stress with similar ketamine doses used in the present study; however, some studies reported reduced oxidative stress following ketamine applications.^36,37^ In this study, hippocampal MDA levels increased insignificantly in the ketamine-applied group, and it was concluded that the applied dose did not significantly affect oxidative stress.

Studies reported a wide range of morphologic alterations with ketamine applications in the spinal cord and the brain.^38,39^ In this study, histopathologic examinations revealed no significant morphological changes in the VEH, MET, FLU, and pCPA-applied groups. On the other hand, ketamine-applied groups, including the ketamine–fluoxetine combination, had significant histopathologic changes, including perineural and perivascular edema, satellitosis, and necrosis. Similar histologic differences and alterations were reported in the literature.^40,41^ These results indicate that ketamine causes neurodegeneration that could not be prevented by antiserotonergic molecules used in this study.

This study revealed the involvement of the serotonergic system in ketamine-induced emotional memory functions, specifically in acquisition and consolidation processes. Increased serotonergic transmission seems to contribute to the drug’s memory-impairing effects. We conclude that ketamine impairs emotional memory and disrupts neuronal integrity through serotonergic mechanisms, as the deterioration is evident, especially when ketamine is combined with other serotonergic enhancers.

Ketamine holds the potential for novel therapeutic indications. However, further investigations are necessary to unravel the precise molecular and cellular pathways that mediate cognitive decline. Continued research into ketamine’s interactions with neurotransmitter systems and their relationship with memory processes may increase our understanding of the effects and side effects of ketamine.
